# Eosinophilic Esophagitis in Children in North-Eastern Poland

**DOI:** 10.3390/jcm9123869

**Published:** 2020-11-28

**Authors:** Katarzyna Zdanowicz, Magdalena Kucharska, Maria Elzbieta Sobaniec-Lotowska, Dariusz Marek Lebensztejn, Urszula Daniluk

**Affiliations:** 1 Department of Pediatrics, Gastroenterology, Hepatology, Nutrition and Allergology, Medical University of Bialystok, 15-274 Bialystok, Poland; magda_leonczak@wp.pl (M.K.); lebensztejn@hoga.pl (D.M.L.); urszula.daniluk@umb.edu.pl (U.D.); 2 Department of Medical Pathomorphology, Medical University of Bialystok, 15-274 Bialystok, Poland; mariasl@umb.edu.pl

**Keywords:** eosinophilic esophagitis, esophagitis, eosinophil, allergy, dysphagia, esophagogastroduodenoscopy, children, Poland

## Abstract

Background: An increase in the incidence of eosinophilic esophagitis worldwide is being observed in children. The aim of the study was to analyze the incidence, clinical manifestations, biochemical markers and endoscopic features of children with eosinophilic esophagitis in comparison to patients with non-eosinophilic esophagitis. Methods: This single-center retrospective study included newly diagnosed children with eosinophilic (EoE) and non-eosinophilic (non-EoE) esophagitis based on endoscopic and histopathological results between January 2013 and December 2018. Result: Among 433 of enrolled children with esophagitis, 36 (8.31%) were diagnosed with EoE (median age of 10 years). Male predominance and an increased percentage of allergy cases in the EoE group were noticed. Dysphagia was the only symptom that significantly differentiated both groups (*p* = 0.006). Endoscopic findings with relevant relationships with EoE included linear fissuring, decreased vascular pattern, trachealization and whitish exudates. No significant difference in the prevalence of other reported diseases between groups was observed. Conclusion: The results of EoE analysis in children from North-Eastern Poland did not differ from reports from other countries. The reported symptoms were not specific for EoE, and only dysphagia and some endoscopic lesions were helpful to differentiate children with EoE from non-EoE.

## 1. Introduction

Eosinophilic esophagitis (EoE) is a chronic disease confined to the esophagus and characterized by an abnormal immune response. It is clinically manifested as an anomalous function of the esophagus and infiltration of inflammatory cells, composed mainly of eosinophils assessed in histopathological examination of mucosal biopsies. The minimum number of eosinophils in the esophageal mucosa necessary for EoE diagnosis is 15 per high-power field (hpf) [1,2]. The inflammatory changes in the esophagus wall are progressive and can lead to fibrous reconstruction and stricture. The pathogenesis of the disease is not fully understood and may involve environmental and genetic factors [1,2]. In the pediatric population, EoE is the second most common cause of chronic or recurrent esophageal dysfunction after gastroesophageal reflux disease (GERD) [3]. In children, EoE often coexists with allergy [1,4]; however, co-occurrences with other inflammatory disorders have also been suggested [1]. Clinical manifestation in children depends on age and consists of gastroesophageal reflux-like symptoms, vomiting, abdominal pain, dysphagia and food impaction [1,2]. Endoscopic changes of the esophagus observed in the pediatric population include stacked circular rings (“feline esophagus”), strictures (particularly proximal strictures), attenuation of the sub-epithelial vascular pattern, linear furrows, whitish papules (representing eosinophil micro-abscesses) and a small caliber of the esophagus [1]. The incidence of EoE is constantly increasing in both adults and children based on recent data [1,5]. This finding can be partly explained by better disease detection due to adherence to biopsy guidelines, which leads to an increase in esophageal biopsies and more detailed histological evaluation of samples [1,6]. Some reports have suggested that environmental factors such as diet and allergens may have the main impact on EoE pathogenesis in children [7]. EoE is diagnosed worldwide in children, and there are only a few available studies concerning EoE manifestation in children and co-occurrence with other diseases [1,2]. Because our department was the only center in the north-eastern part of Poland performing endoscopic procedures in children in recent years, we analyzed the incidence, symptoms, biochemical markers and endoscopic features in children with EoE compared to patients with non-eosinophilic esophagitis (non-EoE) in our region.

## 2. Materials and Methods

This retrospective analysis involved 4177 children with symptoms related to esophageal dysfunction who had esophagogastroduodenoscopies with esophageal biopsies between 2013 and 2018. The patients’ clinical and laboratory data were collected from the hospitals’ documents. All patients underwent the standardized esophagogastroduodenoscopy protocol, which included esophageal biopsies. The same pathologist analyzed all samples. The eosinophils in the most densely infiltrated area were counted under hpf. EoE histopathological criteria from 2017 required at least 15 eosinophils per hpf [1]. To be diagnosed with EoE, children had to meet all the criteria published in the guidelines in 2017 and 2018 [1,2]. Patients were excluded from the analysis if they were already treated due to EoE, had other causes of esophageal eosinophilia (hypereosinophilic syndrome, infection, drug hypersensitivity, connective tissue disorder) or presented a prominent eosinophilic infiltrate in gastric or duodenal biopsies. On admission to the hospital, parents/guardians filled in a standard symptom questionnaire, and then the information was completed by physicians and included in the patients’ medical records. The diagnosis of allergy was made by an allergy specialist based on medical records and verified by a serum-specific IgE panel with food and inhalant allergens, or by skin prick test or skin patch test. Based on the clinical presentation and final histologic result, the patients were divided into two groups with eosinophilic (study group) or non-eosinophilic esophagitis (control group). Statistical analysis was performed using STATISTICA 13.3 software (TIBCO Software Inc., Palo Alto, CA, USA). Chi-2 test and Mann–Whitney U were used to compare eosinophilic esophagitis children with non-eosinophilic esophagitis children. A statistical significance was noticed with *p* value < 0.05. The study was approved by the local Ethics Committee.

## 3. Results

**Figure 1:**
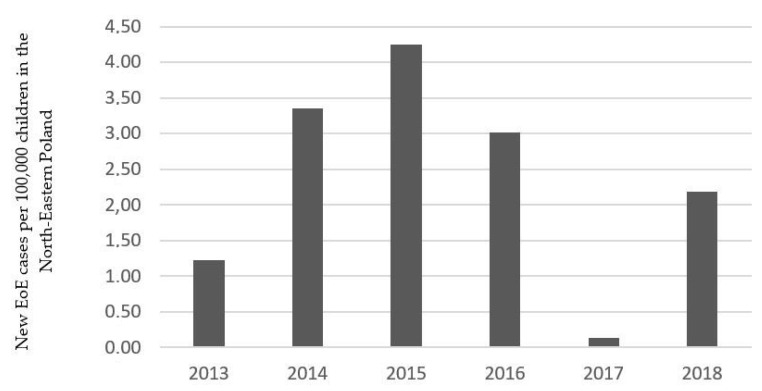
New eosinophilic esophagitis (EoE) cases per 100,000 children in the North-Eastern Poland.

Among 433 children with esophagitis, 36 were diagnosed with EoE (study group) and 397 were classified as a control group (non-EoE). The incidence of EoE in our region was not constant, with a peak in 2015 (4.25 cases per 100,000 children) and the lowest rate in 2017 (0.13 cases per 100,000 children) as is shown on Figure 1. The EoE average annual incidence during the study period was 2.83 cases per 100,000 children. The demographic details, reported symptoms and coexisting diseases in the analyzed groups are listed in Table 1. The median age of children with EoE (10 years, range 2–17 years) was lower than that of those without EoE (13 years, range 1–18 years). A male predominance was evident in the study group (77.78% vs. 49.62%, *p* = 0.002). The patients’ history analysis revealed a higher percentage of allergy cases in children with EoE than in non-EoE subjects, both seasonal (23.08% vs. 8.82%, *p* = 0.002) and food (11.1% vs. 2.52%, *p* = 0.005). There was no significant difference in the prevalence of other reported diseases between groups (Table 1). All patients from the EoE group reported some symptoms; however, only dysphagia was more often observed in children with EoE compared to non-EoE cases (22.2% vs. 7.3%, *p* = 0.02). Regarding age and gender, there were no differences in symptoms in the EoE group (Table S1 Supplementary Materials). The blood assessment showed an increased rate of eosinophilia in children with EoE than in the control group (55.56% vs. 6.55%, *p* < 0.001) (Table 2). No significant differences in other blood results were found between groups. According to the latest guidelines, EoE and GERD can coexist in the same patient; therefore, pH monitoring was performed [1,2]. Abnormal gastroesophageal reflux in 24-h esophageal pH monitoring (*n* = 130) was observed in 29.41% of patients with EoE compared to 69.03% of children from the non-EoE group (*p* = 0.004). Additional test results are presented in Table 2. Among the endoscopy findings, the longitudinal furrowing (*p* < 0.001), decreased vascular pattern (*p* < 0.001), trachealization (*p* = 0.02) and whitish exudates (*p* < 0.001) were the most typical lesions for EoE diagnosis in the children studied (Table 2). Interestingly, normal esophageal mucosa was found in 4 (11.11%) patients from the study group. The probability of EoE diagnosis was higher when two or more lesions were found in the esophagus during endoscopic procedures. No strictures among children with EoE were noted. The median number of eosinophil/hpf in the EoE group was 22 (range 15–45) and 0 (range 0–12) in the non-EoE group. The frequency of *Helicobacter pylori* infection, based on positive result of a urease test and histology examination, was similar in children with EoE (44.44%) and without EoE (45.59%).

## 4. Discussion

Despite the reported worldwide increase in the incidence of EoE, in our study, the incidence of this disease was not constant during the previous years. The average annual incidence rate of EoE for the period between 2013 and 2018 was 2.83 cases per 100,000 children in North-Eastern Poland. A similar result was reported in New Zealand (2.12 per 100,000 children) [8]. However, in Utah, United States of America (USA), the average annual incidence was 24 per 100,000 children [9], in contrast to Serbia, where it was 0.85 per 100,000 children [10]. In a systematic review with a meta-analysis, the overall incidence of EoE was 5.1 (1.5–10.9) per 100,000 children/year and varied significantly between studies. A higher incidence rate was recorded in North America than in Europe, but regional differences were calculated by combining studies conducted on adults and children due to a low number of available pediatric data in Europe. The time of the studies was also a factor influencing the frequency of EoE. The incidence was higher in studies conducted in 2008 and after than before 2008. The various reported incidences of EoE in children are an effect of the different methodology used in studies and the availability for endoscopic procedure necessary for diagnosis [5]. Male dominance among patients with EoE has been already reported and a similar result was noted in our study [11,12]. It has been hypothesized that single nucleotide polymorphisms (SNP) in the thymic stromal lymphopoietin (TSLP) gene and nonsynonymous SNP in the TSLP receptor may be associated with EoE only in male patients [13].

EoE clinical manifestation varies depending on the age of patients. In early childhood, symptoms are related to feeding problems and vomiting. With age, more often abdominal pain, dysphagia and food impaction are observed [1]. In our study, no differences in reported symptoms were noted between age groups, and abdominal pain was the most often recorded complaint in all study groups. Abdominal pain seemed to be one of the main symptoms in the pediatric population with EoE, based on other observations [14,15]. However, dysphagia was the only symptom distinguishing children with EoE from non-EoE patients in our analysis. Interestingly, dysphagia was found to be the main reported symptom in two pediatric studies [8,16]. Despite the occurrence of symptoms that hindered food intake such as abdominal pain, lack of appetite, vomiting or nausea, we did not observe a difference in the prevalence of failure to thrive between both groups. Interestingly, also no abnormalities in growth parameters were found in Mehta et al.’s study assessing the nutritional status of children with EoE [17]. Although food bolus impaction is the main manifestation of EoE in adults, in our study, none of the patients from the EoE group experienced this symptom, similar to reports from Brazil and New Zealand [8,15]. However, a comparable frequency of food bolus impaction in children and adults with EoE (36% vs. 42%) has also been reported [10].

Allergy is one of the most often coexisting diseases with EoE, although the number of concomitant atopic disorders is variable in different studies [1]. A total of 59% of children with EoE and IgE-mediated food allergy was reported in Colorado, USA [17], compared with 29% of children enrolled in a multicenter European study [18]. Interestingly, this association was less frequently observed in our study. Partially, this could be explained by the older age of children diagnosed with EoE in our group compared to children from Colorado. Sensitization to food occurs mainly in younger children IgE [19,20]. Regarding the seasonal allergy, a study from San Diego, CA, USA, showed 47% cases of asthma and 40% of allergic rhinitis among EoE children [14]. Similar results were noted in Texas, USA [21]. On the other hand, the coexistence of asthma and allergic rhinitis in children with EoE in Slovenia was 36% and 4%, respectively [22]. Again, our analysis revealed a lower percentage of seasonal allergy, probably due to a lower prevalence of asthma and allergic rhinitis in children from our region compared to other parts of Poland based on the latest report [23,24]. Recently, an existing association between the onset of EoE symptoms and the peak level of grass pollen has been reported [25].

According to the latest guidelines, no relationships of EoE with celiac disease and inflammatory bowel disease (IBD) were confirmed [1]. In our study, we noted more cases of EoE with concomitant celiac disease or IBD than in other reports—however, without a significant difference in the incidence of these diseases compared to the non-EoE group [26,27]. A higher percentage of children with concomitant celiac disease or IBD in our study group may be associated with some local factors such as the prevalence of diseases in the pediatric population and environmental impact. There are also conflicting data on the protective role of *Helicobacter pylori* infection in the development of EoE. An analysis carried out in Germany showed an inverse relationship between *Helicobacter pylori* infection and EoE incidence [28], contrary to the results of Molina-Infante et al. [29]. In our study, a similar incidence of this infection was detected in both groups.

Differentiation between EoE and GERD can be challenging due to the similarity of symptoms and the presence of eosinophils in the esophageal biopsy [30]. Previously, both diseases were considered distinct entities, but recent data have shown increasing evidence of the coexistence of EoE and GERD [2]. In our study, diagnosis of pathological esophageal reflux based on pH monitoring was confirmed in almost 30% of patients with EoE compared to 20% of cases reported from Serbia [10]. Such a high percentage of GERD may be associated with an influence of enhanced eosinophils infiltration in the esophagus mucosa on the structure and function of the esophagus [1].

The current guidelines recommend performing upper endoscopy with esophageal biopsy to diagnose EoE [1,2]. While microscopic assessment is key, in some cases, macroscopic changes may lead to initial diagnosis. Abnormalities in endoscopic examination may be divided into inflammatory features (edema, exudate, furrowing) often observed in children and fibro-stenotic features (rings, stricture) more common for adults [31,32]. Regardless of which region of the world, similar endoscopic changes are reported among children with EoE [8,10,12,14,15,31]. However, it should be known that a visually normal esophagus does not exclude the diagnosis of EoE. In a group of 381 children diagnosed with EoE in Pennsylvania, USA, about one third of the normal macroscopic image of the esophagus was observed [33]. Similar results were obtained in a study conducted in New Zealand [8]. An even larger percentage of patients (45%) with normal mucosae was found in EoE children from Brazil [15]. In our study, a lack of macroscopic abnormalities were reported in 11.11% of children with EoE, and comparable results were published in the Serbian study [10].

No specific blood markers for EoE diagnosis have been found so far. Eosinophilia in peripheral blood has quite often been demonstrated in patients with EoE, but not in each case [34,35]. The majority of our patients with EoE had peripheral eosinophilia compared to children from the control group. Schlag et al. suggested that peripheral blood eosinophils count can serve as a marker for the assessment of the treatment response in adults with EoE [36]. Since invasive procedures are required to diagnose and monitor therapy of EoE, a non-invasive marker to monitor the disease outcome is needed. Several promising non-invasive biomarkers obtained from peripheral blood, oral swab, urine and stool have been studied in EoE children [37]. Immunohistochemistry seems to be a promising option in clinical diagnosis, selection and monitoring of treatment effects [38]. However, none of them have been recommended for clinical practice so far [1].

The main limitation of the study is the low number of enrolled patients, due to the low number of newly diagnosed children per year in our region. Another limitation is a retrospective analysis. The strength of this study is that our department was the main pediatric endoscopic center with histopathological examination in the north-eastern part of Poland diagnosing all EoE in children in recent years. However, we could not rule out that individual pediatric residents of our region could be diagnosed in other parts of our country.

## 5. Conclusions

The results of EoE analysis in children from North-Eastern Poland did not differ from reports from other countries. The reported symptoms were not specific for EoE, and only dysphagia and some endoscopic lesions were helpful in differentiating children with EoE from non-EoE.

## Figures and Tables

**Table 1:** Demographic data and clinical manifestation in eosinophilic esophagitis (EoE) and non-EoE groups.

	EoE Group	Non-EoE Group	*p* Value
Number of patients	36 (8.31%)	397 (91.7%)	-
Age (year)	10 (2–17)	13 (1–18)	<0.001
Male (*n*)	28 (77.78%)	197 (49.62%)	0.002
BMI			
<10 percentile	10 (27.78%)	73 (18.39%)	NS
>90 percentile	3 (8.33%)	46 (11.59%)	NS
**Co-existing conditions**			
Food allergy	4 (11.11%)	10 (2.52%)	0.02
Seasonal allergy	9 (23.08%)	35 (8.82%)	0.005
Diabetes mellitus type 1	0 (0%)	1 (0.25%)	NA
Celiac disease	4 (11.11%)	28 (6.30%)	NS
IBD	4 (11.11%)	23 (5.79%)	NS
**Symptoms**			
Abdominal pain (*n*)	25 (69.4%)	297 (74.81%)	NS
Failure of thrive (*n*)	11 (30.6%)	76 (19.14%)	NS
Dysphagia (*n*)	8 (22.2%)	29 (7.30%)	0.006
Halitosis (*n*)	7 (19.4%)	51 (12.85%)	NS
Vomiting (*n*)	6 (16.7%)	89 (22.42%)	NS
Lack of appetite (*n*)	6 (16.7%)	54 (13.60%)	NS
Heartburn (*n*)	1 (2.78%)	42 (10.58%)	NS
Nausea (*n*)	3 (8.33%)	65 (16.37%)	NS
Weight loss (*n*)	2 (5.56%)	45 (11.34%)	NS
Eructation (*n*)	3 (8.33%)	55 (13.85%)	NS
Regurgitation (*n*)	2 (5.56%)	9 (2.28%)	NS
Chest pain (*n*)	0 (0%)	10 (2.52%)	NA

BMI- Body Mass Index; IBD- inflammatory bowel disease; NA—not applicable; NS—not significant.

**Table 2:** Endoscopy findings, histology and laboratory results in EoE and non-EoE groups.

	EoE	Non-EoE	*p*
**Endoscopy and histology findings**			
Longitudinal furrowing (*n*)	25 (69.4%)	19 (4.76%)	<0.001
Decrease vascular pattern (*n*)	11 (30.6%)	18 (4.53%)	<0.001
Whitish exudates (*n*)	7 (19.4%)	9 (2.37%)	<0.001
Esophageal erosion (*n*)	5 (13.89%)	40 (10.08%)	NS
Mucosal esophageal erythema (*n*)	4 (11.11%)	41 (10.33%)	NS
Papules/plaques (*n*)	4 (11.11%)	46 (11.59%)	NS
Trachealization/rings (*n*)	3 (8.33%)	5 (1.26%)	0.02
Esophageal polyp (*n*)	1 (2.78%)	6 (1.51%)	NS
Esophageal hernia (*n*)	3 (8.33%)	36 (9.07%)	NS
*Helicobacter pylori* infection (*n*)	16 (44.44%)	181 (45.59%)	NS
Median eosinophil counts (range)	22 (15–45)	0 (0–12)	-
**Number of endoscopic changes:**			
0	4 (11.11%)	249 (65.70%)	<0.001
1	10 (27.78%)	91 (24.01%)	NS
2	14 (38.89%)	43 (11.35%)	<0.001
3	7 (19.44%)	10 (2.52%)	<0.001
**Laboratory results**			
Eosinophilia (*n*) (>400 eos/μL)	20 (55.56%)	26 (6.55%)	<0.001
Hb (median, min-max) (g/dL)	13.05 (10.7–17.0)	13.30 (8.1–17.2)	NS
CRP (median, min-max) (mg/dL)	0.2 (0.0–4.8)	0.2(0.0–52.1)	NS
PLT (median, min-max) (×10^3^/μL)	253 (152–439)	266 (86–643)	NS

CRP—C-reactive protein; Hb—hemoglobin, PLT—platelet; NA—not applicable; NS-not significant.

## Supplementary Materials

The following are available online at https://www.mdpi.com/2077-0383/9/12/3869/s1, Table S1: Clinical symptoms in children with EoE based on patient age and sex.

## References

[1] Lucendo A.J., Molina-Infante J., Arias Á., von Arnim U., Bredenoord A.J., Bussmann C., Dias J.A., Bove M., González-Cervera J., Larsson H. (2017). Guidelines on eosinophilic esophagitis: Evidence-based statements and recommendations for diagnosis and management in children and adults. United Eur. Gastroenterol. J..

[2] Dellon E.S., Liacouras C.A., Molina-Infante J., Furuta G.T., Spergel J.M., Zevit N., Spechler S.J., Attwood S.E., Straumann A., Aceves S.S. (2018). Updated International Consensus Diagnostic Criteria for Eosinophilic Esophagitis: Proceedings of the AGREE Conference. Gastroenterology.

[3] González-Cervera J., Lucendo A.J. (2016). Eosinophilic Esophagitis: An Evidence-Based Approach to Therapy. J. Investig. Allergol. Clin. Immunol..

[4] González-Cervera J., Arias Á., Redondo-González O., Cano-Mollinedo M.M., Terreehorst I., Lucendo A.J. (2017). Association between atopic manifestations and eosinophilic esophagitis: A systematic review and meta-analysis. Ann. Allergy Asthma Immunol..

[5] Arias Á., Pérez-Martínez I., Tenías J.M., Lucendo A.J. (2016). Systematic review with meta-analysis: The incidence and prevalence of eosinophilic oesophagitis in children and adults in population-based studies. Aliment. Pharmacol. Ther..

[6] Syed A.A., Andrews C.N., Shaffer E., Urbanski S.J., Beck P., Storr M. (2012). The rising incidence of eosinophilic oesophagitis is associated with increasing biopsy rates: A population-based study. Aliment. Pharmacol. Ther..

[7] Alexander E.S., Martin L.J., Collins M.H., Kottyan L.C., Sucharew H., He H., Mukkada V.A., Succop P.A., Abonia J.P., Foote H. (2014). Twin and family studies reveal strong environmental and weaker genetic cues explaining heritability of eosinophilic esophagitis. J. Allergy Clin. Immunol..

[8] Weerasekera K., Sim D., Coughlan F., Inns S. (2019). Eosinophilic esophagitis incidence in New Zealand: High but not increasing. Clin. Exp. Gastroenterol..

[9] Robson J., O’Gorman M., McClain A., Mutyala K., Davis C., Barbagelata C., Wheeler J., Firszt R., Smith K., Patel R. (2019). Incidence and Prevalence of Pediatric Eosinophilic Esophagitis in Utah Based on a 5-Year Population-Based Study. Clin. Gastroenterol. Hepatol..

[10] Ristic N., Jankovic R., Dragutinovic N., Atanaskovic-Markovic M., Radusinovic M., Stevic M., Ristic M., Ristic M., Milovanovic T. (2019). Diagnosis of Eosinophilic Esophagitis in Children: A Serbian Single-Center Experience from 2010 to 2017. Med. Princ. Pract..

[11] Dellon E.S., Jensen E.T., Martin C.F., Shaheen N.J., Kappelman M.D. (2014). Prevalence of eosinophilic esophagitis in the United States. Clin. Gastroenterol. Hepatol..

[12] Tan L.N., Srivastava S., Teh M., Quak S.H., Aw M.M. (2017). Eosinophilic oesophagitis in children: An uncommon occurrence in a predominantly Chinese population in Singapore. Singap. Med. J..

[13] Sherrill J.D., Gao P.S., Stucke E.M., Blanchard C., Collins M.H., Putnam P.E., Franciosi J.P., Kushner J.P., Abonia J.P., Assa’ad A.H. (2010). Variants of thymic stromal lymphopoietin and its receptor associate with eosinophilic esophagitis. J. Allergy Clin. Immunol..

[14] Aceves S.S., Newbury R.O., Dohil R., Schwimmer J., Bastian J.F. (2007). Distinguishing eosinophilic esophagitis in pediatric patients: Clinical, endoscopic, and histologic features of an emerging disorder. J. Clin. Gastroenterol..

[15] Pinheiro M.I., de Góes Cavalcanti L.P., Honório R.S., de Alencar Moreno L.H., Fortes M.C., da Silva C.A. (2013). Eosinophilic esophagitis in brazilian pediatric patients. Clin. Med. Insights Pediatr..

[16] Gómez Torrijos E., Sánchez Miranda P., Donado Palencia P., Castro Jimenez A., Rodriguez Sánchez J., Mendez Díaz Y., Moreno Lozano L., Garcia Rodriguez R. (2017). Eosinophilic Esophagitis: Demographic, Clinical, Endoscopic, Histologic, and Atopic Characteristics of Children and Teenagers in a Region in Central Spain. J. Investig. Allergol. Clin. Immunol..

[17] Mehta P., Furuta G.T., Brennan T., Henry M.L., Maune N.C., Sundaram S.S., Menard-Katcher C., Atkins D., Takurukura F., Giffen S. (2018). Nutritional State and Feeding Behaviors of Children With Eosinophilic Esophagitis and Gastroesophageal Reflux Disease. J. Pediatr. Gastroenterol. Nutr..

[18] Hoofien A., Dias J.A., Malamisura M., Rea F., Chong S., Oudshoorn J., Nijenhuis-Hendriks D., Otte S., Papadopoulou A., Romano C. (2019). Pediatric Eosinophilic Esophagitis: Results of the European Retrospective Pediatric Eosinophilic Esophagitis Registry (RetroPEER). J. Pediatr. Gastroenterol. Nutr..

[19] Fiocchi A., Pecora V., Petersson C.J., Dahdah L., Borres M.P., Amengual M.J., Huss-Marp J., Mazzina O., Di Girolamo F. (2015). Sensitization pattern to inhalant and food allergens in symptomatic children at first evaluation. Ital. J. Pediatr..

[20] Daniluk U., Alifier M., Kaczmarski M., Stasiak-Barmuta A., Lebensztejn D. (2015). Longitudinal observation of children with enhanced total serum IgE. Ann. Allergy Asthma Immunol..

[21] Hiremath G., Byramji D., Pacheco A., Constantine G., Davis C., Shulman R., Olive A. (2016). Eosinophilic Esophagitis in Children and Its Relationship with Parental Allergies: Texas Children’s Hospital Experience. Dig. Dis. Sci..

[22] Homan M., Blagus R., Jeverica A.K., Orel R. (2015). Pediatric Eosinophilic Esophagitis in Slovenia: Data From a Retrospective 2005-2012 Epidemiological Study. J. Pediatr. Gastroenterol. Nutr..

[23] Samoliński B., Sybilski A.J., Raciborski F., Tomaszewska A., Samel-Kowalik P., Walkiewicz A., Lusawa A., Borowicz J., Gutowska-Ślesik J., Trzpil L. (2009). Prevalence of rhinitis in Polish population according to the ECAP (Epidemiology of Allergic Disorders in Poland) study. Otolaryngol. Pol..

[24] Lipiec A., Sybilski A., Rapiejko P., Furmańczyk K., Namysłowski A., Zieliński W., Malkiewicz M., Bilińska D., Chłopek K., Samoliński B. (2019). Prevalence of allergic rhinitis and asthma in Poland in relation to pollen counts. Adv. Dermatol. Allergol..

[25] Fahey L., Robinson G., Weinberger K., Giambrone A.E., Solomon A.B. (2017). Correlation Between Aeroallergen Levels and New Diagnosis of Eosinophilic Esophagitis in New York City. J. Pediatr. Gastroenterol. Nutr..

[26] Lucendo A.J., Arias Á., Tenias J.M. (2014). Systematic review: The association between eosinophilic oesophagitis and coeliac disease. Aliment. Pharmacol. Ther..

[27] Johnsson M., Bove M., Bergquist H., Olsson M., Fornwall S., Hassel K., Wold A.E., Wennerås C. (2011). Distinctive blood eosinophilic phenotypes and cytokine patterns in eosinophilic esophagitis, inflammatory bowel disease and airway allergy. J. Innate Immun..

[28] von Arnim U., Wex T., Link A., Messerschmidt M., Venerito M., Miehlke S., Malfertheiner P. (2016). Helicobacter pylori infection is associated with a reduced risk of developing eosinophilic oesophagitis. Aliment. Pharmacol. Ther..

[29] Molina-Infante J., Gutierrez-Junquera C., Savarino E., Penagini R., Modolell I., Bartolo O., Prieto-García A., Mauro A., Alcedo J., Perelló A. (2018). Helicobacter pylori infection does not protect against eosinophilic esophagitis: Results from a large multicenter case-control study. Am. J. Gastroenterol..

[30] Kia L., Hirano I. (2015). Distinguishing GERD from eosinophilic oesophagitis: Concepts and controversies. Nat. Rev. Gastroenterol. Hepatol..

[31] Kim H.P., Vance R.B., Shaheen N.J., Dellon E.S. (2012). The prevalence and diagnostic utility of endoscopic features of eosinophilic esophagitis: A meta-analysis. Clin. Gastroenterol. Hepatol..

[32] Gonsalves N. (2014). Distinct features in the clinical presentations of eosinophilic esophagitis in children and adults: Is this the same disease?. Dig. Dis..

[33] Liacouras C.A., Spergel J.M., Ruchelli E., Verma R., Mascarenhas M., Semeao E., Flick J., Kelly J., Brown–Whitehorn T., Mamula P. (2005). Eosinophilic esophagitis: A 10-year experience in 381 children. Clin. Gastroenterol. Hepatol..

[34] Dellon E.S., Gibbs W.B., Fritchie K.J., Rubinas T.C., Wilson L.A., Woosley J.T., Shaheen N.J. (2009). Clinical, endoscopic, and histologic findings distinguish eosinophilic esophagitis from gastroesophageal reflux disease. Clin. Gastroenterol. Hepatol..

[35] Botan V., Dos Santos Borges T.K., Rocha Alves É., Claudino Pereira Couto S., Bender Kohnert Seidler H., Muniz-Junqueira M.I. (2017). Enhanced activation of eosinophils in peripheral blood and implications for eosinophilic esophagitis diagnosis. J. Gastroenterol. Hepatol..

[36] Schlag C., Miehlke S., Heiseke A., Brockow K., Krug A., von Arnim U., Straumann A., Vieth M., Bussmann C., Mueller R. (2015). Peripheral blood eosinophils and other non-invasive biomarkers can monitor treatment response in eosinophilic oesophagitis. Aliment. Pharmacol. Ther..

[37] Hines B.T., Rank M.A., Wright B.L., Marks L.A., Hagan J.B., Straumann A., Greenhawt M., Dellon E.S. (2018). Minimally invasive biomarker studies in eosinophilic esophagitis: A systematic review. Ann. Allergy Asthma Immunol..

[38] Zdanowicz K., Kucharska M., Reszec J., Lebensztejn D.M., Daniluk U. (2020). Immunohistochemical markers for eosinophilic esophagitis. Scand. J. Gastroenterol..

