# Supplementary material for: Eosinophilic Esophagitis in Children in North-Eastern Poland
Source: J Clin Med. 2020 Nov 28;9(12):3869. doi: 10.3390/jcm9123869 (PMC7760083; doi:10.3390/jcm9123869)
Supplement: Supplementary file 1 [file jcm-09-03869-s001.pdf]

Table S1. Clinical symptoms in children with EoE based on patient age and sex.

EoE Group *n* (%)	<10 Years (*n* = 17)	≥10 Years (*n* = 19)	*p* Value	Female (*n* = 8)	Male (*n* = 28)	*p* Value
Abdominal pain	10 (58.82%)	15 (78.95%)	NS	5 (62.50%)	20 (71.43%)	NS
Failure to thrive	6 (35.29%)	5 (26.32%)	NS	0 (0.00%)	11 (39.28%)	NA
Dysphagia	3 (17.65%)	5 (26.32%)	NS	1 (12.50%)	7 (25.00%)	NS
Halitosis	3 (17.65%)	4 (21.05%)	NS	3 (37.50%)	4 (14.29%)	NS
Vomiting	3 (17.65%)	3 (15.78%)	NS	2 (25.00%)	4 (14.29%)	NS
Lack of appetite	2 (11.76%)	4 (21.05%)	NS	1 (12.50%)	5 (17.86%)	NS
Heartburn	0 (0.00%)	1 (5.26%)	NA	0 (0.00%)	1 (3.57%)	NA
Nausea	1 (5.88%)	2 (10.53%)	NS	1 (12.50%)	2 (7.14%)	NS
Weight loss	0 (0.00%)	2 (10.53%)	NA	0 (0.00%)	2 (7.14%)	NA
Eructation	1 (5.88%)	2 (10.53%)	NS	0 (0.00%)	3 (10.71%)	NA
Regurgitation	2 (11.76%)	0 (0.00%)	NA	1 (12.50%)	1 (3.57%)	NS
Chest pain	0 (0.00%)	0 (0.00%)	NA	0 (0.00%)	0 (0.00%)	NA

NA—not applicable; NS—not significant.
